# Changes in Microbiota and Bacterial Protein Caseinolytic Peptidase B During Food Restriction in Mice: Relevance for the Onset and Perpetuation of Anorexia Nervosa

**DOI:** 10.3390/nu11102514

**Published:** 2019-10-18

**Authors:** Manon Dominique, Romain Legrand, Marie Galmiche, Saïda Azhar, Camille Deroissart, Charlène Guérin, Jean-Luc do Rego, Fatima Leon, Séverine Nobis, Grégory Lambert, Nicolas Lucas, Pierre Déchelotte

**Affiliations:** 1 TargEDys SA, University of Rouen Normandy, 76183 Rouen, France; mdominique@targedys.com (M.D.); legrandromain@hotmail.fr (R.L.); mgalmiche@targedys.com (M.G.); saida-az@hotmail.fr (S.A.); camillederoissart@gmail.com (C.D.); glambert@targedys.com (G.L.); lucasnicolas@hotmail.fr (N.L.); 2 Inserm UMR1073, Nutrition, Gut and Brain Laboratory, University of Rouen Normandy, Unirouen, 76183 Rouen, France; charlene.guerin@univ-rouen.fr; 3 Institute for Research and Innovation in Biomedicine (IRIB), University of Rouen Normandy, Unirouen, 76183 Rouen, France; jean-luc.do-rego@univ-rouen.fr (J.-L.d.R.); Fati.jpl@free.fr (F.L.); severine-nobis@orange.fr (S.N.); 4 Animal Behavior Platform, Service Commun d’Analyse Comportementale (SCAC), University of Rouen Normandy, 76183 Rouen, France; 5 Rouen University Hospital, CHU Charles Nicolle, 76183 Rouen, France

**Keywords:** anorexia, food restriction, ClpB, microbiota, *Enterobacteriaceae*

## Abstract

Microbiota contributes to the regulation of eating behavior and might be implicated in the pathophysiology of anorexia nervosa. ClpB (Caseinolytic peptidase B) protein produced mainly by the *Enterobacteriaceae* family has been identified as a conformational mimetic of α-MSH, which could result in similar anorexigenic effects. The aim of this study was to highlight the role of the microbiome and the ClpB protein in deregulation and self-maintenance of anorexia pathology. Male C57Bl/6 mice were undergone to the ABA (Activity-Based Anorexia) protocol: after 5 days of acclimatization, both ABA and LFA (Limited Food Access) mice had progressively limited access to food until D17. At the end of protocol, the plasma ClpB concentration and *Enterobacteriaceae* DNA in colonic content were measured. As expected, dietary restriction induced lost weight in LFA and ABA mice. At D10, colonic permeability and plasma concentration of the ClpB protein were significantly increased in LFA and ABA mice vs. controls. At D17, plasma concentration of ClpB was increased in LFA and ABA mice and, it was correlated with proportion of *Enterobacteriaceae* in the faeces. These abnormally high ClpB concentrations and all associated factors, and therefore might contribute to the initiation and/or perpetuation of anorexia nervosa by interfering with satiety signaling.

## 1. Introduction

Eating Disorders (ED) are public health problems that have continued to worsen in recent years with a prevalence of 3.5% from 2000–2006 to 7.8% in 2013–2018 [1]. Among these disorders, anorexia nervosa (AN) is characterized by a difficulty in maintaining a minimum weight and an obsession with weight and body shape [2], the pathophysiology of which is multifactorial and remains partially debated [3].

Among the proposed mechanisms of AN, the role of the gut microbiota in regulating the physiology of AN is increasingly recognized [4,5]. Indeed, studies have shown that intestinal microbial composition is influenced directly by food, in the short and long term [6,7,8]. Conversely, behavior [9] and appetite [10] are modulated at least in part by several gut-microbiota derived signals, among which bacterial products (e.g., peptides, neurotransmitters) have been shown to influence peripheral and central mechanisms of satiety, reward [11,12] and anxiety [13]. Finally, microbiota composition is implicated in the regulation of body composition: dysbiosis has been reported both in obese individuals [14] and in patients with AN [15]. Moreover, increased *Escherichia coli*, a leading representative of *Enterobacteriaceae* in gut microbiota was also observed in anorexic patients [16]. Altogether, these data strongly suggest that dysfunction of the microbiota-intestine-brain axis in response to exogenous triggering factors might be a key factor in the onset and/or perpetuation of ED [10,17]. Communication between microbiota, gut and brain may rely on various microbiota-derived signals, such as proteins, peptides, monoamines, metabolites, or even gut-produced immunoglobulins gaining access to the brain or modulating afferent neuronal or hormonal regulations generated in the splanchnic area [17]. Among bacterial proteins, ClpB (Caseinolytic peptidase B), a heat shock protein produced by *Enterobacteriaceae* [18] including *E. coli* is of particular relevance to the control of satiety [19] since it holds in common a six amino acid discontinuous epitope sharing molecular mimicry with α-melanocyte-stimulating hormone (α-MSH), the main central neuropeptide signaling satiety in the hypothalamus [20,21]. In addition, other studies have shown that α-MSH could also be found at peripheral level [22]. Moreover, α-MSH could induce the activation of MC4R present on intestinal enteroendocrine L cells [22,23]. Through this specificity, ClpB could stimulate the secretion by enteroendocrine L cells of the satiating hormones GLP-1 or PYY and activate vagal and hormonal pathways leading to hypothalamic activation of the POMC neurons releasing α-MSH [10,11]. In accordance with a role of this protein in the physiological and pathological regulation of eating behavior, ClpB was found naturally in the plasma of healthy subjects and at a higher level in patients with eating disorders [24]. 

In addition to the direct effect of ClpB mentioned previously, the hypothesis that microbial proteins may also modulate eating behavior through the intestinal production of specific immunoglobulins (Ig) can be suggested. Indeed, previous reports have detected Ig which react with α-MSH, in the sera of both healthy individuals and rats [25]. The levels of these Ig correlate with psychological traits characteristic of eating disorders [25]. This suggests that α-MSH reactive Ig may interfere with melanocortin signaling in both normal and pathological conditions. Moreover, a recent study showed that the levels of α-MSH-reactive IgG, the binding of melanocortin 4 receptor (MC4R) and the cellular internalization rate of MC4R-expressing cells were all lower in obese subjects [26]. Inverse results were found in anorexic and bulimic patients [26]. Other studies also confirmed the implication of α-MSH reactive Ig in the physiological regulation of feeding and mood [27]. In patients with eating disorders, increasing ClpB plasma levels correlated with plasma levels of anti-ClpB and anti-α-MSH Ig [19]. These factors emphasize the physiological involvement of anti-α-MSH Ig in the regulation of food intake.

Thus, bacterial ClpB protein appears as a candidate for interfering with endogenous pathway of satiety regulation. To get further insights in its involvement during food restriction, we performed the present study in a well-established model of food restriction in rodents, the Activity-Based Anorexia (ABA) model, and evaluated the impact of food restriction on the plasma ClpB protein and its related Ig and on the proportion of *Enterobacteriaceae*.

## 2. Materils and Methods

### 2.1. Animal Experimentation

Animal experimentation procedures were approved by the Local Ethical Committee of Normandy (approval CENOMEXA n°1112–05). Male C57Bl/6 mice (Janvier Labs, Genest-Saint-Isle, France), at 7 weeks old were kept in holding cages (four mice per cage) at environmental conditions 22 °C ± 3 °C and relative humidity of 40 ± 20% on a 12 h light-dark cycle with lights on at 10:00 a.m. During acclimatization period, all mice were given *ad libitum* access to water and standard food (Kliba Nafag, Germany). 

At D1 of the protocol, all mice were randomized individually into 3 groups: An *ad libitum* group (Control, *n* = 16), a limited-food access group (LFA, *n* = 16) and an activity-based anorexia group (ABA, *n* = 16). ABA mice were placed individually in cages with an activity wheel connected to Running Wheel ^®^ software (Intellibio, Seichamps, France). 

Food access was progressively limited in ABA and LFA groups from 6 h per day at D6, to 3 h at D9 and until the end of the experiment. Mice always had free access to water. Body weight, water and food intake were measured at 9:00 a.m. each day.

At D10, 8 mice of each group were chosen according to their weight and were anaesthetized by ketamine/xylazine (Imalgene^®^ 1000, Murial/Xylazine Rompun 2%, Bayer) intraperitoneally and were euthanized by decapitation. Blood samples were taken from the mesenteric artery before decapitation. The hypothalamus was taken to perform qPCR to analyze the anorexigenic (POMC) and orexigenic (AgRP) neuronal populations. Intracolonic faeces were taken to perform qPCR to analyze the *Enterobacteriaceae* DNA. The plasma was recovered after centrifugation (3000× *g*, 20 min, 4 °C). Samples were taken and stored at −80 °C if their analysis was not done immediately.

At D17, the end of the experiment, remaining mice underwent the same procedures as D10.

### 2.2. Permeability

Colon permeability was assessed by measured FITC-dextran (4 kDa) (Sigma) by Ussing chambers. FITC-dextran (5 mg/mL) was placed on the mucosal side. After 3 h at 37 °C, medium from the serosal side was removed and stored at −80 °C. The fluorescence level of FITC-dextran (excitation at 485 nm, emission at 535 nm) was measured in a 96-well black plate with spectrometer Chameleon V (Hidex, Turku, Finland). Values were converted to concentration (mg/mL) using a concentration standard curve. 

### 2.3. ClpB Concentration

The presence of the protein ClpB was measured by the technique of enzyme linked immunosorbent assay (ELISA) previously described by Breton et al., 2016 [9]. For this, two antibodies were used: rabbit polyclonal anti-ClpB (Delphi Genetics, Brussels, BEL) and a mouse monoclonal antibody anti-ClpB (Delphi Genetics, Brussels, BEL). The optical density was determined at 405 nm using a microplate reader Infinite F50 (Tecan Life Sciences, Switzerland). Each determination was performed in duplicate.

### 2.4. ClpB and α-MSH Ig Assay

Plasma levels of Ig reacting with ClpB or α-MSH were measured using enzyme-linked immunosorbent assay according to a published protocol [28]. For this, a concentration of 2 μg/mL of ClpB protein (Delphi Genetics, Brussels, BEL) or α-MSH peptides (Bachem, Budendorf, Swiss) were used to coat 96-well Maxisorp plates (Nunc, Rochester, NY, USA). Mice plasma samples were diluted at 1:200 in dissociative buffer (3 M NaCl and 1.5 M glycine buffer, pH 8.9) to determine the total Ig levels. Two antibodies were used for detection: Alkaline phosphatase (AP)-conjugated goat anti-mouse IgG or anti-mouse IgM (1:2000) (Jackson ImmunoResearch Laboratories, St. Thomas Place, Ely, UK). The optical density was determined at 405 nm using an Infinite F50 microplate reader (Tecan Life Sciences, Switzerland). Blank optical density values (without the addition of plasma samples) were subtracted from the sample optical density values. Each sample was done in duplicate.

### 2.5. qPCR Assay for Faecal Enterobacteriaceae DNA

*Enterobacteriaceae* DNA in faeces were extracted with the ZymoBIOMICS Kit according to the protocol given by the supplier (ZymoResearch, Irvine, CA, USA). After extraction, the total DNA was quantified using a NanoDrop spectrophotometer (ThermoScientific, Waltham, MA, USA). qPCR was performed on 1 ng/µL of DNA and with Light Cycler^®^480 SYBR^®^ Green I Master (Roche, Swiss). The primers for detection of *Enterobacteriaceae* were: 5′-TGTGCCCAGATGGGATTAGC-3′ and 3′-TTAACCTTGCGGCCGTACTC-5′. The relative quantity of each DNA was calculated using standard curves normalized to a reference *16s DNA* gene. 

### 2.6. RT-qPCR Assay for Hypothalamus Neuronal Populations mRNA

Hypothalamic total RNA was extracted within cold TRIZOL reagent (Invitrogen, Carlsbad, CA, USA). After extraction, the total RNA was quantified using a NanoDrop spectrophotometer (ThermoScientific, Waltham, MA, USA). cDNA was generated by reverse transcription with 1 µg of total RNA using M-MLV Reverse Transcriptase (200 U/µL) (ThermoFisher, Waltham, MA, USA). RT-qPCR was performed on all samples using a BioRad CFX96 Real Time PCR System (BioRad, Hercules, CA, USA) and SYBR Green Master Mix (Life Technologies, Carlsbad, CA, USA). The primers for detection of *pomc* were: 5′-CCTCCTGCTTCAGACCTCCA-3′ and 5′-GGCTGTTCATCTCCGTTGC-3′; for *agrp*, 5′-GCAGACCGAGCAGAAGAT-3′ and 5′-CTGTTGTCCCAAGCAGGA-3′. The relative quantity of each mRNA was calculated from standard curves, normalized to a reference *gapdh* gene.

### 2.7. Statistical Analysis

Data are shown as means +/− standard error of means (SEM). Before statistical analysis the normality was evaluated by the Kolmogorov-Smirnov test. Then, statistical significance was calculated by the unpaired *t*-test, one-way ANOVA or two-way ANOVA, as appropriate. All statistical calculations were performed using Prism 6.0 software (GraphPad Software, Inc., San Diego, CA, USA) and *p* < 0.05 was considered as significant.

## 3. Results

### 3.1. Body Weight and Food Intake

During the adaptation phase (D1–D6), the animals had the weight between 20 g and25 g. From the beginning of the dietary restriction (D6), the animals started to lose weight. The ABA and LFA mice lost significant weight compared to the control (** *p* < 0.01, D7, D8). This difference in weight loss continues until the end of the experiment (D17) (*** *p* < 0.001, D9 to D17). From D10 until the end of endurance, ABA mice lost significantly more weight than the LFA mice (* *p* < 0.05, D10, D12; ** *p* < 0.01, D13 to D17) (Supplementary data, Figure S1A).

Food intake (Supplementary data, Figure S1B) of ABA mice increased between D4 and D6 as compared to LFA and the control mice (*** *p* < 0.001) (Supplementary data, Figure S1B), (*** *p* < 0.001, D6) (Supplementary data, Figure S1C). However, since the beginning of the limited access to food (D7), food intake decreased significantly in all groups (*** *p* < 0.001) (Supplementary data, Figure S1B,C). At D10, when access time to food is shortest, food intake was significantly reduced compared to D6 (before restriction) (*** *p* < 0.001) with a reduction of 34% in the LFA group and 58% in the ABA group (Supplementary data, Figure S1C).

From D11, restriction was even intensified for the ABA group compared to the LFA group (* *p* < 0.05, D12; ** *p* < 0.01, D13; *** *p* < 0.001, D15) (Supplementary data, Figure S1B) until the end of this experiment (reduction of 16.4%) (Supplementary data, Figure S1C).

### 3.2. Wheel Activity

Total wheel activity increased during the restriction phase (D6–D10) of ABA mice. (Supplementary data, Figure S2A,B) as compared to the adaptation phase (D2–D5) which resulted mainly from an increased activity during the dark phase. (Supplementary data, Figure S2C,D). From D9, wheel activity decreased during the dark phase (Supplementary data, Figure S2C,D), while it increased during the light phase (Supplementary data, Figure S2E,F) vs. D6–D10.

### 3.3. Intestinal Permeability, ClpB and Immunoglobulins Plasma Levels

**Figure 1:**
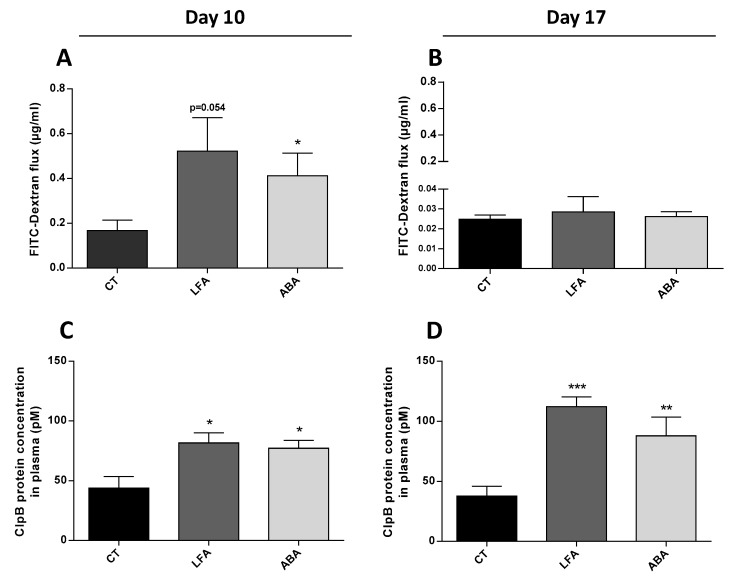
Intestinal permeability measure and ClpB concentration in plasma. The intestinal permeability was measured by an ELISA assay after FITC-dextran passage in the ussing chamber (**A**) at D10 and (**B**) at D17. The ClpB concentration was measured in plasma in pM by an ELISA assay (**C**) at D10 and (**D**) at D17. Data are means ± SEM. Unpaired Mann-Whitney test (**A**, *p* = 0.0541) or ne-way ANOVA test with Holm-Sidak’s post-tests (**C**,**D**); *** *p* < 0.001, ** *p* < 0.01, * *p* < 0.05.

Intestinal permeability assessed in vitro by FITC-dextran flux increased at D10 (Figure 1A) while no difference was observed at D17 (Figure 1B).

ClpB protein concentration in plasma increased significantly at D10 (Figure 1C) and D17 (Figure 1D) in ABA and LFA groups vs. controls.

**Figure 2:**
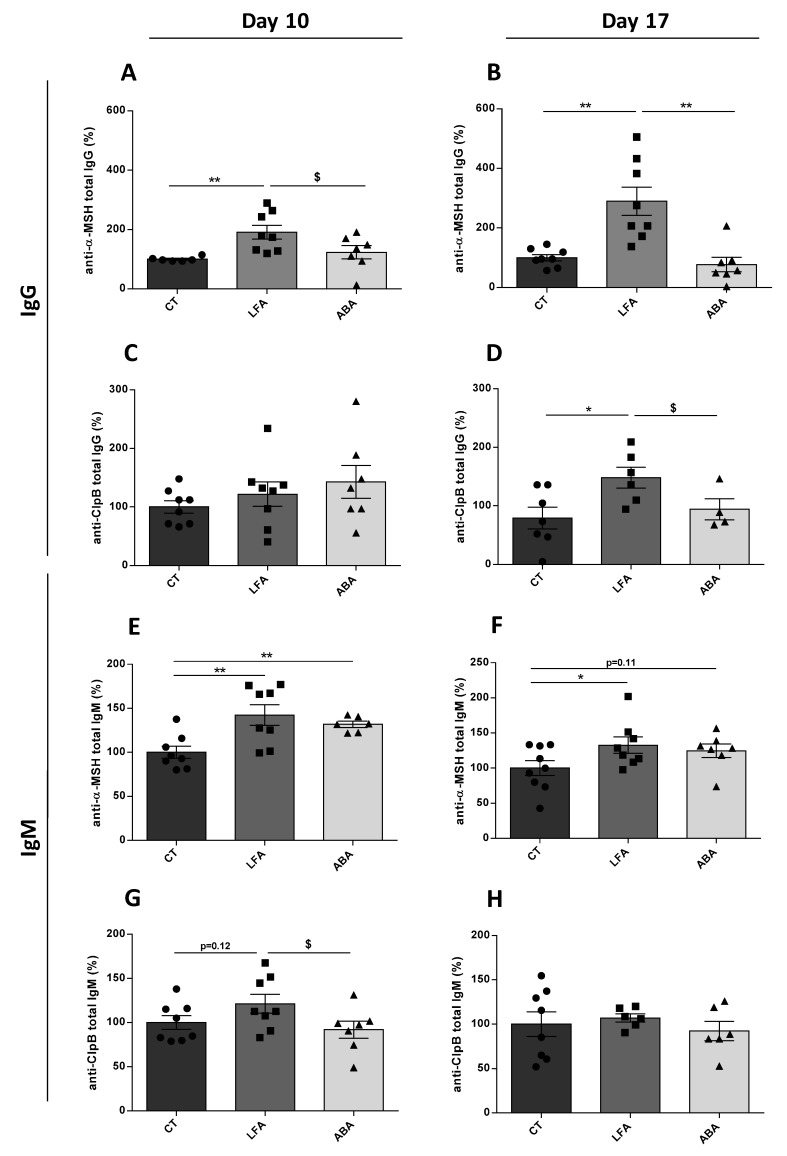
Impact of food restriction and physical activity on modulation of anti-α-MSH and anti-ClpB IgG and IgM. Anti-α-MSH and anti-ClpB IgG antibodies (%) (**A**,**C**) at D10 and (**B**,**D**) at D17 were measured in plasma. Anti-α-MSH and anti-ClpB IgM were measured in the same way at (**E**,**G**) at D10 and (**F**,**H**) at D17 in plasma. Data are means ± SEM. Unpaired Mann-Whitney test (**D**,**F**) or unpaired *t*-test (**A**,**B**,**E**,**G**,**H**); ** *p* < 0.01, * *p* < 0.05, ^$^ *p* < 0.10.

The plasma levels of anti-α-MSH IgG were increased at D10 and D17 in LFA vs. controls and the ABA group (Figure 2A,B). IgM anti-α-MSH levels were increased at D10 and D17 in LFA and ABA vs. controls (Figure 2E,F). The anti-ClpB IgG were increased at D17, but not at D10 in LFA vs. controls and ABA group (Figure 2C,D). The anti-ClpB IgM were increased at D10 but not at D17 in LFA vs. controls and vs. ABA group at D10 (Figure 2G,H).

### 3.4. Faecal Enterobacteriaceae DNA

**Figure 3:**
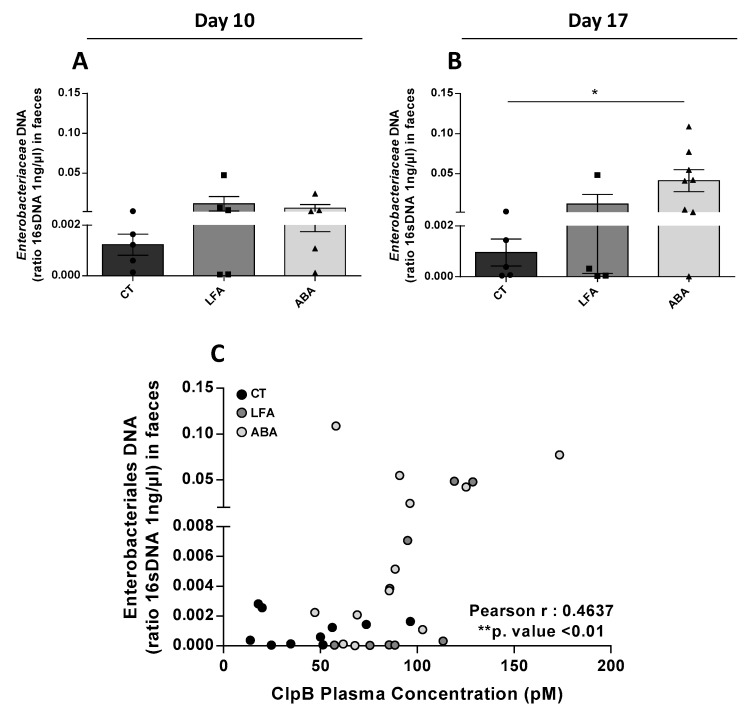
*Enterobacteriaceae* DNA in faeces and correlation with ClpB Plasma concentration. Relative quantitative expression of *Enterobacteriaceae* DNA in faeces by qPCR (**A**) at D10 and (**B**) at D17. The relative expression was calculated with a 1 ng/µL *Enterobacteriaceae* concentration normalized by *16sDNA* gene. (**C**) Correlation between *Enterobacteriaceae* DNA in faeces and ClpB plasma concentration (pM). Data are means ± SEM. Unpaired Mann-Whitney test (**A**,**B**) or Pearson correlation (**C**); ** *p* < 0.01, * *p* < 0.05.

Relative quantitative amount of *Enterobacteriaceae* DNA in faeces was not different at D10 (Figure 3A) but increased at D17 (Figure 3B). This increased in *Enterobacteriaceae* DNA is positively correlated with the ClpB plasma concentration (Figure 3C).

### 3.5. Hypothalamic Neuropeptides

**Figure 4:**
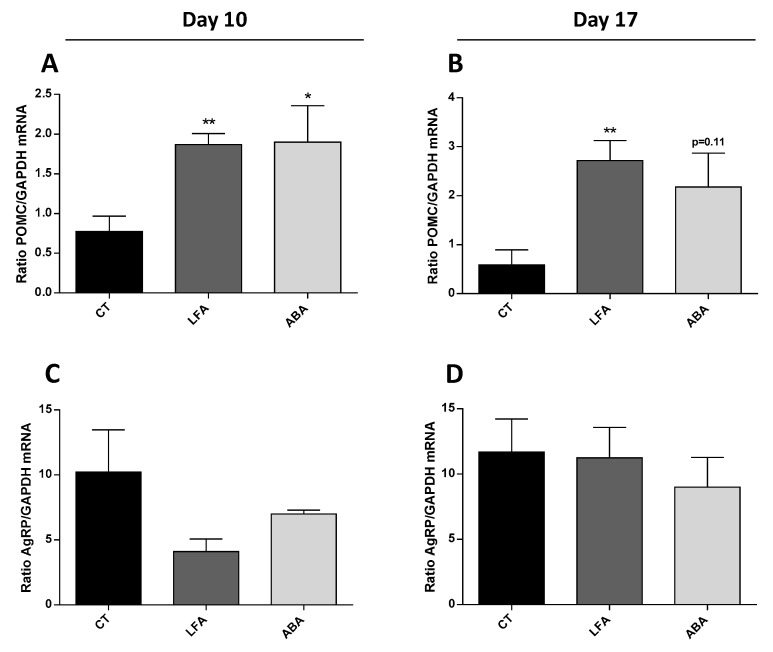
Impact of food restriction on neuronal population gene expression. Relative quantitative expression of (**A**,**B**) POMC and (**C**,**D**) AgRP mRNA in the hypothalamus by qPCR. The relative abundance of mRNA was calculated as the ratio of the normalized level (SQ of gene of interest mRNA/SQ of GAPDH mRNA). Data are means ± SEM. Unpaired *t*-test (**A**,**B**); ** *p* < 0.01, * *p* < 0.05.

Hypothalamic POMC mRNA relative expression increased in LFA and ABA groups at D10 (Figure 4A) and D17 (Figure 4B). AgRP mRNA relative expression was not altered at either D10 (Figure 4C) or D17 (Figure 4D).

## 4. Discussion

In this study, we highlighted that an increase of ClpB plasma concentration correlated with the relative amount of *Enterobacteriaceae* in faeces of food restricted mice. As expected in the ABA model, mice significantly lost weight which was amplified by the wheel activity of ABA mice [29,30]. Reduced food intake in ABA was not a consequence of wheel activity, since activity ceased when food was again available, which rather suggests that the hyperactivity was a consequence of food restriction.

Several studies have previously reported an increase permeability in the colon of ABA mice [31,32] which suggests that a dysfunction of the intestinal barrier may occur during anorexia nervosa. In the present study, the increased intestinal permeability observed at D10 was associated with an increase in the plasma concentration of the ClpB protein. Accordingly, a previous study from our group [33] reported alterations of the colonic mucosa proteome in ABA mice, suggesting that a decreased energy supply to the colonic mucosa may compromise its functional integrity metabolism [33]. 

No difference in intestinal permeability or ClpB levels were observed between the LFA and ABA groups. This suggests that physical activity alone has no significant effect on colonic barrier function in this model, and that food restriction induces the increase of ClpB protein plasma level. This increased ClpB may result from an increased transcellular passage of this protein across the enterocytes. In fact, the enterocyte endocytosis of intact proteins is a well-established process. Milk proteins such as β-lactoglobulin (18.36 kDa) and α-lactalbumin (14.2 kDa) can cross the enterocytes via a non-specific liquid phase endocytosis mechanism and reach the basolateral side by a transcytosis mechanism [34]. Even larger proteins such as the 44 kDa glycoprotein Horseradish Peroxidase (HRP) can enter the intestinal absorptive cells by apical endocytosis [35]. Thus, endocytosis of the whole 96 kDa ClpB looks plausible. 

Alternatively, paracellular passage may be possible for lower molecular weight proteins or fragments. An increased paracellular passage may be allowed by a degradation of the intercellular tight junction proteins network (e.g., occludin, claudin-1) [36], as already reported in inflammatory bowel diseases [37,38], irritable bowel syndrome [39,40], obesity [41] and malnutrition states [42] including the ABA model [31], and several intestinal diseases [43]. This paracellular pathway may be of relevance for the fragments of ClpB. Indeed, the ClpB protein has a capacity to fragment naturally as shown in vitro (Mogk et al., 1999), and smaller fragments may access the basolateral space before finally reaching the plasma compartment. This hypothesis is consistent with the increased ex vivo colonic passage of the FITC Dextran molecule (4 kDa) observed in food restricted mice. Our home-made ELISA test probably identified both the whole ClpB protein and several of its fragments.

In the present study, we also observed that food restriction induced changes in the plasma levels of anti-α-MSH IgG and IgM. The most consistent finding was an increase of anti-α-MSH IgG and IgM in the LFA group at D10 and D17. This immune activation may result from the activation of the hypothalamo-pituitary axis with the increased release of CRF and related-peptides such as α-MSH [44], and consequently an increase in the corresponding Ig. Our results suggest a higher increase of anti-α-MSH Ig as compared to anti-ClpB Ig. This may reflect the fact that the primary antibodies used for the ELISA assay may recognize other epitopes in the α-MSH structure in addition to those in common with ClpB. Furthermore, this increase could be a consequence of food restriction, with or without activity, which is stressful for mice. A previous study showed that repeated exposure of rats to mild stress induced by food restriction and repeated blood sampling increased the levels and affinity of α-MSH reactive IgG Ig [27]; passive transfer of these Ig purified from the blood of stressed to naïve animals induced acute food intake and suppressed anxiety. This suggests that the production of these Ig might be an adaptive response to stress aiming to counteract its effects by blunting the satiating effect of α-MSH. The moderate increase of anti-α-MSH Ig in the ABA group raises the question of a possible immunosuppressive effect caused by intense physical activity, which remains debated [45]. Although other studies need to be done, these results confirm the hypothesis proposed by Fetissov et al. that the anorexia physiopathology performed from altered signaling between the gut microbiota, the immune system and the neuropeptides involved in feeding behavior regulation [46].

In our study, colonic content analysis showed an increase proportion of *Enterobacteriaceae* DNA in the ABA group at D17 compared to controls, with intermediate values in the LFA group. This is in accordance with the increased ClpB plasma level at D17, since *Enterobacteriaceae* are known to produce the ClpB protein [18,24]. Already at D10 an increased production and/or release of ClpB by *Enterobacteriaceae* may have occurred before a significant growth of this family. Accordingly, the *Enterobacteriaceae* DNA correlated with ClpB plasma concentration across groups. Moreover, the significant correlation between the ClpB protein plasma levels and the relative amount of *Enterobacteriaceae* seen only in the anorexic mice suggests that dietary restriction impairs microbiota composition. The increased *Enterobacteriaceae* and ClpB protein production is in accordance with previous papers reporting increased *Enterobacteriaceae* in patients with anorexia and in malnourished animals [15]. Under critical dietary restriction conditions, an increased production of ClpB may also be an adaptive process to support the survival of the microorganism since ClpB is a chaperone protein [47]. Accordingly, Breton et al. reported that the production of ClpB by in vitro *E. coli* was increased in the stationary growth phase, after the disposal of added nutrients during the exponential phase [11]. The food restriction led to an increase in *Enterobacteriaceae* population, combined with an increase in both ClpB production and colonic permeability, together these factors have led to abnormally high plasma levels of ClpB. 

The increased plasma concentration of ClpB or its fragments allows a direct central effect at the hypothalamic level of stimulating the POMC-related satiating pathways [11] which in turn contribute to either the onset or the perpetuation of anorexia and hyperactivity which could be explain by a satiating and anxiogenic effects of ClpB mimicking α-MSH [19]. 

**Figure 5:**
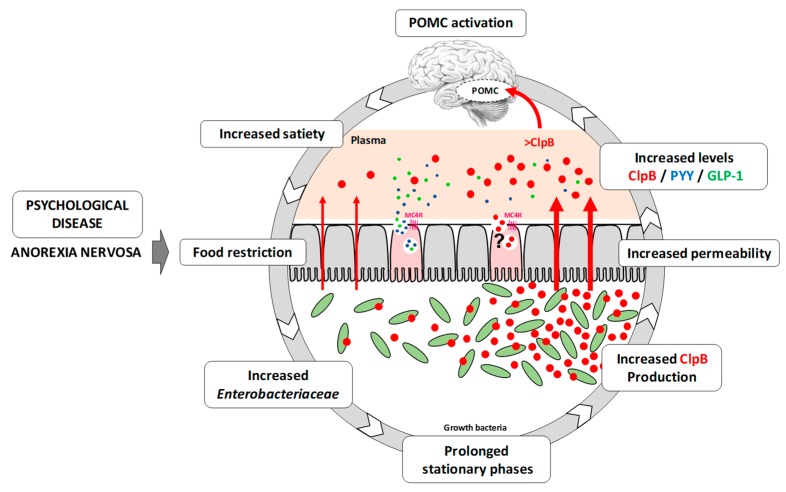
Vicious circle of the physiopathology of anorexia nervosa. Anorexia is characterized by psychological disorders (deformation of the self-image, obsessive fear of gaining weight) which are the cause of a restriction of food intake. This limited dietary intake leads to dysbiosis, characterized by an increase in *Enterobacteriaceae* within the microbiota. This increase generates an increased production of the ClpB protein, resulting from the prolongation of the stationary growth phase of these bacteria. In parallel, dietary restriction also causes an increase in intestinal permeability, which explains the increased passage of this protein through the intestinal mucosa. This protein is then found in the bloodstream with the other satietogenic peptides (GLP-1, PYY) released via the activation of the MC4R receptor present on the L cells. The mechanism of passage of this protein through the mucosa remains unknown, but hypotheses suggest that it may pass through the mucosa in fragments or through a mechanism of endocytosis. Finally, because of its anorectic action, the ClpB protein can activate anorexigenic neuronal populations such as POMC, whose response will lead to an increase in satiety. As well, the vicious cycle of the physiology of anorexia nervosa will can continue...

It is important to emphasize that this biological approach of anorexia nervosa does not come in contradiction with the well-established triggering role of psychological stress. Indeed, stress might reduce food intake at the hypothalamic level or via the mesocorticolimbic system, but also at the peripheral level by increasing intestinal permeability [48,49] and altering microbiota virulence, proliferation and release of pro-inflammatory and anorexigenic signals acting on neuronal afferents [50,51]. Using the data from this article and others, we can propose anintegrative perspective (Figure 5) which links dietary restriction, stress, microbiota-gut-brain axis dysregulation (including increased ClpB signaling) and the on-going self-maintenance of anorexia nervosa. When fully confirmed, this approach may open innovative therapeutic perspectives via modulation of gut microbiota by different nutritional, microbial or pharmacological approaches [52,53].

## 5. Conclusions

In conclusion, we have shown here that bacterial ClpB plasma levels increase during dietary restriction in mice, regardless of physical activity, and correlates with amount of *Enterobacteriaceae* in feces. This brings additional arguments for the role of the gut microbiota in the mechanisms of eating disorders and so, suggests its impact in the perpetuation and self-maintenance of the anorexia. These data suggest that nutritional or probiotic interventions aiming to restore gut microbiota may be useful in the therapeutic strategy of eating disorders.

## Supplementary Materials

The following are available online at https://www.mdpi.com/2072-6643/11/10/2514/s1, Figure S1: Food restriction model confirmation–Body weight and Food intake, Figure S2: ABA model confirmation–Wheel activity.

## References

[1] Galmiche M., Déchelotte P., Lambert G., Tavolacci M.P. (2019). Prevalence of eating disorders over the 2000–2018 period: A systematic literature review. Am. J. Clin. Nutr..

[2] American Psychiatric Association (2013). DSM–5.

[3] Gorwood P., Blanchet-Collet C., Chartrel N., Duclos J., Dechelotte P., Hanachi M., Fetissov S., Godart N., Melchior J.-C., Ramoz N. (2016). New Insights in Anorexia Nervosa. Front. Neurosci..

[4] Kleiman S.C., Carroll I.M., Tarantino L.M., Bulik C.M. (2015). Gut feelings: A role for the intestinal microbiota in anorexia nervosa?. Int. J. Eat. Disord..

[5] Roubalová R., Procházková P., Papežová H., Smitka K., Bilej M., Tlaskalová-Hogenová H. (2019). Anorexia nervosa: Gut microbiota-immune-brain interactions. Clin. Nutr. Edinb. Scotl..

[6] David L.A., Maurice C.F., Carmody R.N., Gootenberg D.B., Button J.E., Wolfe B.E., Ling A.V., Devlin A.S., Varma Y., Fischbach M.A. (2014). Diet rapidly and reproducibly alters the human gut microbiome. Nature.

[7] Wu G.D., Chen J., Hoffmann C., Bittinger K., Chen Y.-Y., Keilbaugh S.A., Bewtra M., Knights D., Walters W.A., Knight R. (2011). Linking long-term dietary patterns with gut microbial enterotypes. Science.

[8] Singh R.K., Chang H.-W., Yan D., Lee K.M., Ucmak D., Wong K., Abrouk M., Farahnik B., Nakamura M., Zhu T.H. (2017). Influence of diet on the gut microbiome and implications for human health. J. Transl. Med..

[9] Dinan T.G., Cryan J.F. (2017). Microbes, Immunity, and Behavior: Psychoneuroimmunology Meets the Microbiome. Neuropsychopharmacology.

[10] Van de Wouw M., Schellekens H., Dinan T.G., Cryan J.F. (2017). Microbiota-Gut-Brain Axis: Modulator of Host Metabolism and Appetite. J. Nutr..

[11] Breton J., Tennoune N., Lucas N., Francois M., Legrand R., Jacquemot J., Goichon A., Guérin C., Peltier J., Pestel-Caron M. (2016). Gut Commensal E. coli. Proteins Activate Host Satiety Pathways following Nutrient-Induced Bacterial Growth. Cell Metab..

[12] Strandwitz P. (2018). Neurotransmitter modulation by the gut microbiota. Brain Res..

[13] Lach G., Schellekens H., Dinan T.G., Cryan J.F. (2018). Anxiety, Depression, and the Microbiome: A Role for Gut Peptides. Neurother. J. Am. Soc. Exp. Neurother..

[14] Castaner O., Goday A., Park Y.-M., Lee S.-H., Magkos F., Shiow S.-A.T.E., Schröder H. (2018). The Gut Microbiome Profile in Obesity: A Systematic Review. Int. J. Endocrinol..

[15] Breton J., Déchelotte P., Ribet D. (2019). Intestinal microbiota and Anorexia Nervosa. Clin. Nutr. Exp..

[16] Million M., Angelakis E., Maraninchi M., Henry M., Giorgi R., Valero R., Vialettes B., Raoult D. (2013). Correlation between body mass index and gut concentrations of Lactobacillus reuteri, Bifidobacterium animalis, Methanobrevibacter smithii and Escherichia coli. Int. J. Obes..

[17] Fetissov S.O. (2017). Role of the gut microbiota in host appetite control: Bacterial growth to animal feeding behaviour. Nat. Rev. Endocrinol..

[18] Legrand R., Lucas N., Dominique M., Azhar S., Deroissart C., Le Solliec M.-A., Rondeaux J., Nobis S., Guérin C., Léon F. (2019). Commensal Hafnia alvei strain reduces food intake and fat mass in obese mice-a new potential probiotic for appetite and body weight management. Int. J. Obes..

[19] Tennoune N., Chan P., Breton J., Legrand R., Chabane Y.N., Akkermann K., Järv A., Ouelaa W., Takagi K., Ghouzali I. (2014). Bacterial ClpB heat-shock protein, an antigen-mimetic of the anorexigenic peptide α-MSH, at the origin of eating disorders. Transl. Psychiatry.

[20] Kim M.S., Rossi M., Abusnana S., Sunter D., Morgan D.G., Small C.J., Edwards C.M., Heath M.M., Stanley S.A., Seal L.J. (2000). Hypothalamic localization of the feeding effect of agouti-related peptide and alpha-melanocyte-stimulating hormone. Diabetes.

[21] Guan H.-Z., Dong J., Jiang Z.-Y., Chen X. (2017). α-MSH Influences the Excitability of Feeding-Related Neurons in the Hypothalamus and Dorsal Vagal Complex of Rats. BioMed Res. Int..

[22] Manning S., Batterham R.L. (2014). Enteroendocrine MC4R and energy balance: Linking the long and the short of it. Cell Metab..

[23] Panaro B.L., Tough I.R., Engelstoft M.S., Matthews R.T., Digby G.J., Møller C.L., Svendsen B., Gribble F., Reimann F., Holst J.J. (2014). The melanocortin-4 receptor is expressed in enteroendocrine L cells and regulates the release of peptide YY and glucagon-like peptide 1 in vivo. Cell Metab..

[24] Breton J., Legrand R., Akkermann K., Järv A., Harro J., Déchelotte P., Fetissov S.O. (2016). Elevated plasma concentrations of bacterial ClpB protein in patients with eating disorders. Int. J. Eat. Disord..

[25] Fetissov S.O., Harro J., Jaanisk M., Järv A., Podar I., Allik J., Nilsson I., Sakthivel P., Lefvert A.K., Hökfelt T. (2005). Autoantibodies against neuropeptides are associated with psychological traits in eating disorders. Proc. Natl. Acad. Sci. USA.

[26] Lucas N., Legrand R., Bôle-Feysot C., Breton J., Coëffier M., Akkermann K., Järv A., Harro J., Déchelotte P., Fetissov S.O. (2019). Immunoglobulin G modulation of the melanocortin 4 receptor signaling in obesity and eating disorders. Transl. Psychiatry.

[27] Sinno M.H., Do Rego J.C., Coëffier M., Bole-Feysot C., Ducrotté P., Gilbert D., Tron F., Costentin J., Hökfelt T., Déchelotte P. (2009). Regulation of feeding and anxiety by alpha-MSH reactive autoantibodies. Psychoneuroendocrinology.

[28] Fetissov S.O. (2011). Neuropeptide autoantibodies assay. Methods Mol. Biol..

[29] Lewis D.Y., Brett R.R. (2010). Activity-based anorexia in C57/BL6 mice: Effects of the phytocannabinoid, Delta9-tetrahydrocannabinol (THC) and the anandamide analogue, OMDM-2. Eur Neuropsychopharmacol..

[30] Nobis S., Goichon A., Achamrah N., Guérin C., Azhar S., Chan P., Morin A., Bôle-Feysot C., do Rego J.C., Vaudry D. (2018). Alterations of proteome, mitochondrial dynamic and autophagy in the hypothalamus during activity-based anorexia. Sci. Rep..

[31] Jésus P., Ouelaa W., François M., Riachy L., Guérin C., Aziz M., Do Rego J.-C., Déchelotte P., Fetissov S.O., Coëffier M. (2014). Alteration of intestinal barrier function during activity-based anorexia in mice. Clin. Nutr. Edinb. Scotl..

[32] Achamrah N., Nobis S., Breton J., Jésus P., Belmonte L., Maurer B., Legrand R., Bôle-Feysot C., do Rego J.L., Goichon A. (2016). Maintaining physical activity during refeeding improves body composition, intestinal hyperpermeability and behavior in anorectic mice. Sci. Rep..

[33] Nobis S., Achamrah N., Goichon A., L’Huillier C., Morin A., Guérin C., Chan P., do Rego J.L., do Rego J.C., Vaudry D. (2018). Colonic Mucosal Proteome Signature Reveals Reduced Energy Metabolism and Protein Synthesis but Activated Autophagy during Anorexia-Induced Malnutrition in Mice. Proteomics.

[34] Caillard I., Tomé D. (1995). Transport of beta-lactoglobulin and alpha-lactalbumin in enterocyte-like Caco-2 cells. Reprod. Nutr. Dev..

[35] Blok J., Mulder-Stapel A.A., Ginsel L.A., Daems W.T. (1981). Endocytosis in absorptive cells of cultured human small-intestinal tissue: Horseradish peroxidase, lactoperoxidase, and ferritin as markers. Cell Tissue Res..

[36] Buckley A., Turner J.R. (2018). Cell Biology of Tight Junction Barrier Regulation and Mucosal Disease. Cold Spring Harb. Perspect. Biol..

[37] Poritz L.S., Garver K.I., Green C., Fitzpatrick L., Ruggiero F., Koltun W.A. (2007). Loss of the tight junction protein ZO-1 in dextran sulfate sodium induced colitis. J. Surg. Res..

[38] Xu P., Elamin E., Elizalde M., Bours P.P.H.A., Pierik M.J., Masclee A.A.M., Jonkers D.M.A.E. (2019). Modulation of Intestinal Epithelial Permeability by Plasma from Patients with Crohn’s Disease in a Three-dimensional Cell Culture Model. Sci. Rep..

[39] Bertiaux-Vandaële N., Youmba S.B., Belmonte L., Lecleire S., Antonietti M., Gourcerol G., Leroi A.-M., Déchelotte P., Ménard J.-F., Ducrotté P. (2011). The expression and the cellular distribution of the tight junction proteins are altered in irritable bowel syndrome patients with differences according to the disease subtype. Am. J. Gastroenterol..

[40] Cheng P., Yao J., Wang C., Zhang L., Kong W. (2015). Molecular and cellular mechanisms of tight junction dysfunction in the irritable bowel syndrome. Mol. Med. Rep..

[41] Brun P., Castagliuolo I., Leo V.D., Buda A., Pinzani M., Palù G., Martines D. (2007). Increased intestinal permeability in obese mice: New evidence in the pathogenesis of nonalcoholic steatohepatitis. Am. J. Physiol.Gastrointest. Liver Physiol..

[42] Genton L., Cani P.D., Schrenzel J. (2015). Alterations of gut barrier and gut microbiota in food restriction, food deprivation and protein-energy wasting. Clin. Nutr..

[43] Bischoff S.C., Barbara G., Buurman W., Ockhuizen T., Schulzke J.-D., Serino M., Tilg H., Watson A., Wells J.M. (2014). Intestinal permeability-a new target for disease prevention and therapy. BMC Gastroenterol..

[44] Dhillo W.S., Small C.J., Seal L.J., Kim M.-S., Stanley S.A., Murphy K.G., Ghatei M.A., Bloom S.R. (2002). The Hypothalamic Melanocortin System Stimulates the Hypothalamo-Pituitary-Adrenal Axis in vitro and in vivo in Male Rats. Neuroendocrinology.

[45] Nieman D.C., Nehlsen-Cannarella S.L. (1991). The Effects of Acute and Chronic Exercise on Immunoglobulins. Sports Med..

[46] Fetissov S.O., Hökfelt T. (2019). On the origin of eating disorders: Altered signaling between gut microbiota, adaptive immunity and the brain melanocortin system regulating feeding behavior. Curr. Opin. Pharmacol..

[47] Lee S., Sowa M.E., Watanabe Y., Sigler P.B., Chiu W., Yoshida M., Tsai F.T.F. (2003). The structure of ClpB: A molecular chaperone that rescues proteins from an aggregated state. Cell.

[48] Vanuytsel T., van Wanrooy S., Vanheel H., Vanormelingen C., Verschueren S., Houben E., Salim Rasoel S., Tόth J., Holvoet L., Farré R. (2014). Psychological stress and corticotropin-releasing hormone increase intestinal permeability in humans by a mast cell-dependent mechanism. Gut.

[49] Wallon C., Yang P.-C., Keita A.V., Ericson A.-C., McKay D.M., Sherman P.M., Perdue M.H., Söderholm J.D. (2008). Corticotropin-releasing hormone (CRH) regulates macromolecular permeability via mast cells in normal human colonic biopsies in vitro. Gut.

[50] Biaggini K., Borrel V., Szunerits S., Boukherroub R., N’Diaye A., Zébré A., Bonnin-Jusserand M., Duflos G., Feuilloley M., Drider D. (2017). Substance P enhances lactic acid and tyramine production in Enterococcus faecalis V583 and promotes its cytotoxic effect on intestinal Caco-2/TC7 cells. Gut Pathog..

[51] Biaggini K., Barbey C., Borrel V., Feuilloley M., Déchelotte P., Connil N. (2015). The pathogenic potential of Pseudomonas fluorescens MFN1032 on enterocytes can be modulated by serotonin, substance P and epinephrine. Arch. Microbiol..

[52] Lam Y.Y., Maguire S., Palacios T., Caterson I.D. (2017). Are the Gut Bacteria Telling Us to Eat or Not to Eat? Reviewing the Role of Gut Microbiota in the Etiology, Disease Progression and Treatment of Eating Disorders. Nutrients.

[53] Dominique M., Breton J., Guérin C., Bole-Feysot C., Lambert G., Déchelotte P., Fetissov S. (2019). Effects of Macronutrients on the In Vitro Production of ClpB, a Bacterial Mimetic Protein of α-MSH and Its Possible Role in Satiety Signaling. Nutrients.

