# Supplementary material for: Changes in Microbiota and Bacterial Protein Caseinolytic Peptidase B During Food Restriction in Mice: Relevance for the Onset and Perpetuation of Anorexia Nervosa
Source: Nutrients. 2019 Oct 18;11(10):2514. doi: 10.3390/nu11102514 (PMC6835841; doi:10.3390/nu11102514)
Supplement: Supplementary file 1 [file nutrients-11-02514-s001.pdf]

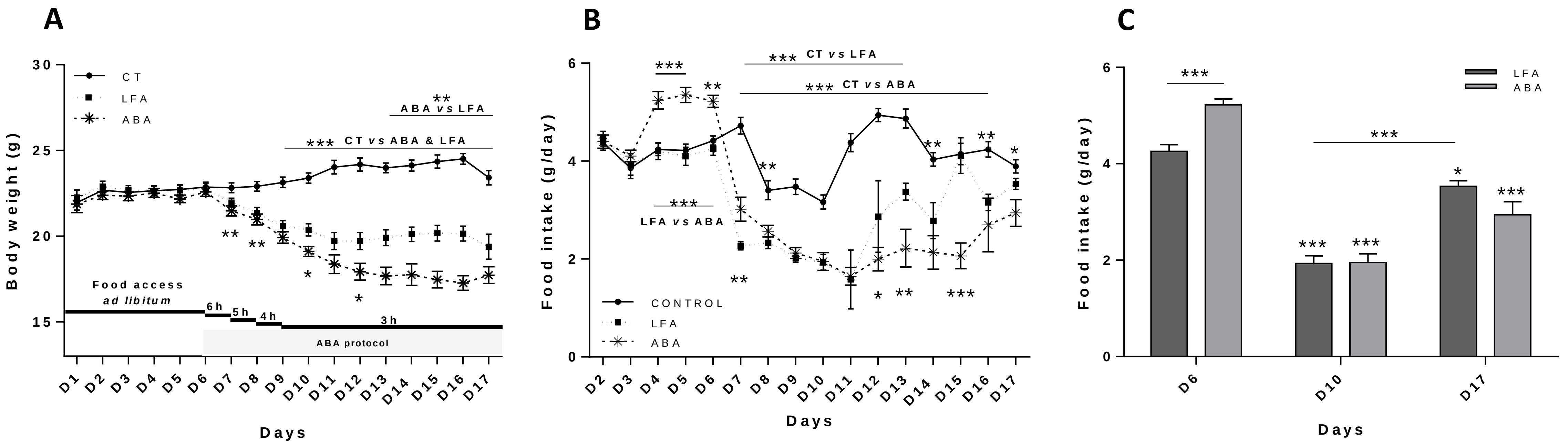
A
B
C
Body weight (g)
Food intake (g/day)
Days
CT
LFA
ABA
CONTROL
CT vs ABA & LFA
ABA vs LFA
CT vs LFA
CT vs ABA
LFA vs ABA
Food access
ad libitum
6 h
5 h
4 h
3 h
ABA protocol

Figure S1

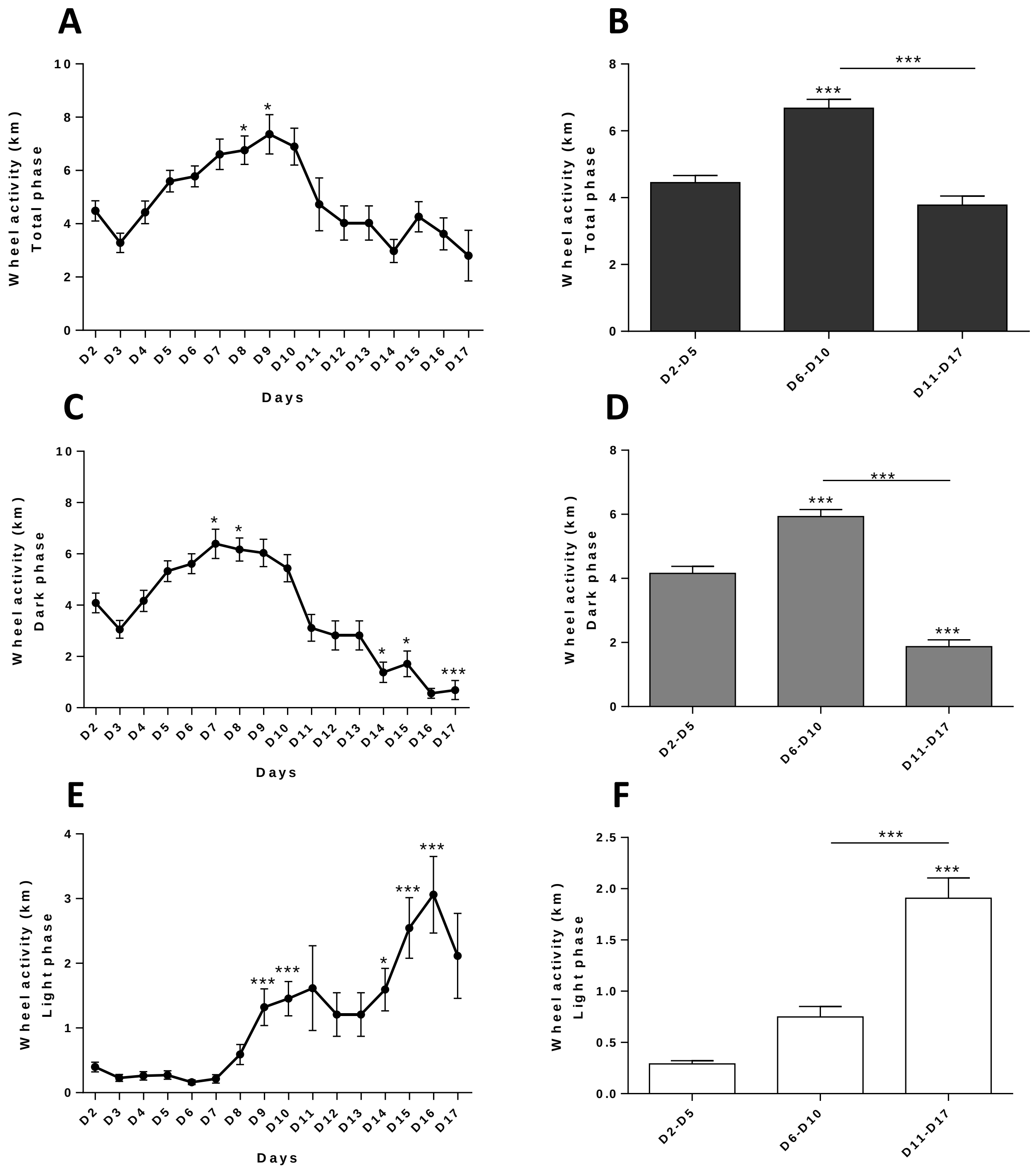
A
B
C
D
E
F
Wheel activity (km)
Total phase
Dark phase
Light phase
Days
D2 D3 D4 D5 D6 D7 D8 D9 D10 D11 D12 D13 D14 D15 D16 D17
D2-D5
D6-D10
D11-D17
***
*

Figure S2

FIGURE LEGENDS

Figure S1. Food restriction model confirmation – Body weight and Food intake. (A) Body weight (g) and (B and C) Food Intake (g/day) measured throughout the experiment. D0 to D6, all mice have food access *ad libitum*; D6, D7, D8, D9 to D17, food access was limited at 6h, 5h, 4h and 3h respectively for LFA and ABA groups. n=16 at D10 et n=8 at D17. Data are means ± SEM. Two-way ANOVA test with Tukey's post-test (A, B, C); ***p<0.001, **p<0.01, *p<0.05.

Figure S2. ABA model confirmation – Wheel activity. ABA Mice wheel activity expressed (km) was measured everyday using RunningWheel® software (Intellibio). Activities measured (A, B) during the total phase, (C, D) the dark phase and (E, F) the light phase. n=16. Data are means ± SEM. Kruskal-Wallis test with Dunns' post-tests (A, B, C, D, E, F); ***p<0.001, *p<0.05.
